# Radiation dose of chaperones during common pediatric computed tomography examinations

**DOI:** 10.1007/s00247-020-04681-6

**Published:** 2020-05-15

**Authors:** Daniel Overhoff, Meike Weis, Philipp Riffel, Sonja Sudarski, Matthias F. Froelich, Peter Fries, Stefan Schönberg, Joshua Gawlitza

**Affiliations:** 1 Institute of Clinical Radiology and Nuclear Medicine, University Medical Center Mannheim, Medical Faculty Mannheim, Heidelberg University, Heidelberg, Germany; 2 Clinic of Diagnostic and Interventional Radiology, Saarland University Medical Center, Kirrberger Straße – Gebäude 50.1, Homburg, 66421 Germany

**Keywords:** Children, Computed tomography, Dosimetry, Imaging protocols, Radiation dose reduction, Radiation exposure

## Abstract

**Background:**

One main challenge in pediatric imaging is to reduce motion artifacts by calming young patients. To that end, the Radiological Society of North America (RSNA) as early as 1997 stated the necessity of adults accompanying their child during the child’s examination. Nonetheless, current research lacks data regarding radiation dose to these chaperones.

**Objective:**

The aim of this study was to measure the radiation dose of accompanying adults during state-of-the-art pediatric CT protocols.

**Materials and methods:**

In addition to a 100-kV non-contrast-enhanced chest CT (Protocol 1), we performed a 70-kV contrast-enhanced chest protocol (Protocol 2) using a third-generation dual-source CT. We acquired data on the radiation dose around the scanner using digital dosimetry placed right at the gantry, 1 m away, as well as beside the gantry. We acquired the CT-surrounding radiation dose during scanning of a pediatric phantom as well as 12 pediatric patients.

**Results:**

After conducting 10 consecutive phantom scans using Protocol 1, we found the location with the highest cumulative dose acquired was right next to the gantry opening, at 3 μSv. Protocol 2 showed highest cumulative dose of 2 μSv at the same location. For Protocol 1, the location with the highest radiation doses during pediatric scans was right next to the gantry opening, with doses of 0.75±0.70 μSv. For Protocol 2, the highest radiation was measured 1 m away at 0.50±0.60 μSv. No radiation dose was measured at any time beside the gantry.

**Conclusion:**

Our results provide proof that chaperones receive low radiation doses during state-of-the-art CT examinations. Given knowledge of these values as well as the optimal spots with the lowest radiation doses, parents as well as patients might be more relaxed during the examination.

## Introduction

As the number of pediatric CT examinations rises, more non-pediatric-specialized hospitals are challenged with pediatric imaging [1]. Special protocols need to be applied because children are more susceptible to ionizing radiation and subsequent risk of neoplasia [2, 3]. Tin-filtering and other low-dose imaging approaches combined with iterative reconstruction noise reduction algorithms can lead to a significant dose reduction [4].

Children often have to be immobilized to reduce motion artifacts [5]. This can intensify the negative experience of the young patients. To make the examination as comfortable as possible and thereby reduce potential motion artifacts, calming the children is one of the main goals during the procedure, and to this end leaving parents with the children during the examination is often indispensable [6]. As recommended, parents have to wear a lead gown to reduce radiation [7]. Further, an assessment of the radiation dose monitored by digital dosimetry is mandatory. As stated in the latest overhaul of the German radiation protection law, this digitally measured radiation has to be recorded [8]. These changes in guidelines underline the necessity of research in the area of radiation doses in adults accompanying children during their CT examination.

Our aim was to examine the radiation dose received by chaperones during typical pediatric chest CT protocols. In addition to a scan of a chest phantom, we measured radiation doses during actual pediatric scans using the mandatory digital dosimetry.

## Materials and methods

### Study design

The study protocol, which is in accordance with the Declaration of Helsinki, was approved by the local ethics committee. To get an initial impression of the parental radiation doses, we scanned a soft-tissue phantom (20-cm diameter, fat-tissue isodense) with a circular cut-out (15-cm diameter) mimicking the child’s chest, multiple times. In the second phase, we used digital dosimetry to measure the radiation dose around the scanner during 12 routine pediatric scans. All parents were asked to consent to the radiation dose acquisition during the scan.

### Computed tomography protocols

All CT examinations were performed on a third-generation dual-source CT (SOMATOM Force; Siemens Healthineers, Forchheim, Germany) without sedation and without breathing commands. As previously described, a dedicated pediatric body positioning aid device was used in children shorter than 120 cm, with the device fixing the body in place with arms above the head. The device was not used in children taller than 120 cm. An 80-kVp/34-mA topogram was performed in all children to minimize the z-axis scan range.

Two protocols were examined in this study (Table 1). The scanning parameters for the 100-kV chest non-contrast protocol (Protocol 1) were as follows: 100 reference mA using automated tube current modulation (Care Dose 4D; Siemens Healthineers), 0.25-s gantry rotation time, pitch 3.2, 192×0.6-mm detector collimation. This protocol included the use of a dedicated 0.6-mm tin filter adjacent to the source as previously described [9]. Additionally, we used a contrast-enhanced protocol. The contrast-enhanced chest protocol at 70 kVp (Protocol 2) had parameters as follows: 64 reference mAs using automated tube voltage modulation with a reference kilovoltage of 70 (Care kV; Siemens Healthineers), pitch 3.2, 192×0.6-mm detector collimation.

**Table 1:** Computed tomography parameters

	Protocol 1	Protocol 2
Contrast	Chest, non-enhanced	Chest, contrast-enhanced
Detector rows	2×192	2×192
Tube voltage (kVp)	100	70
Reference tube current (mAs)	100	64
Rotation time (s)	0.25	0.25
Pitch	3.2	3.2
Collimation (mm)	192×0.6	192×0.6
Tin filtration (mm)	0.6	None

Contrast medium was diluted in a 50–50 ratio, using 1 mL iodine-based contrast agent (Iomeron 400; Bracco Imaging, Milan, Italy) per kilogram body weight. The bolus was applied manually during a fixed delay of 25 s, prior to the scan. Thereby, a mixed contrast phase was achieved. All images were postprocessed using iterative reconstruction techniques (ADMIRE; Siemens Healthineers).

### Radiation dose assessment

**Fig. 1:**
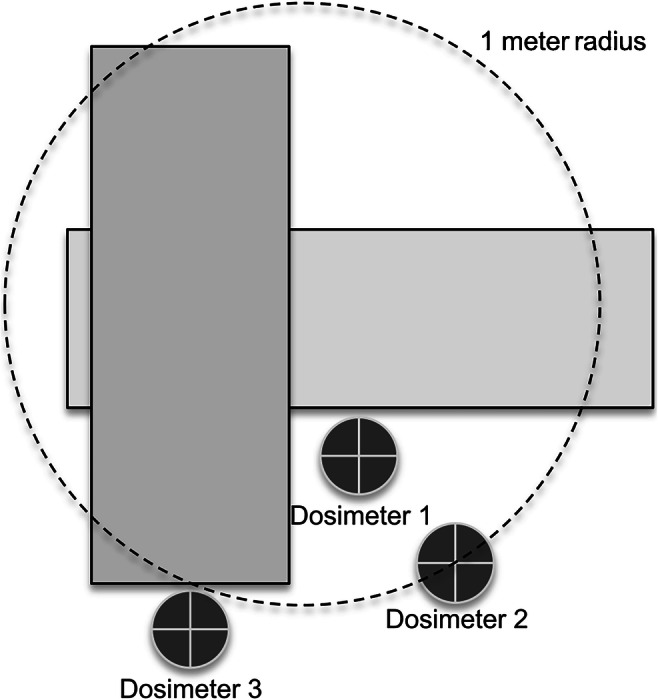
Diagram shows the locations of the three dosimeters next to the CT scanner. One was right next to table and gantry opening (Dosimeter 1); the second was in the diagonal prolongation, 1 m away (Dosimeter 2); and the last was beside the gantry (Dosimeter 3)

We measured radiation dose using digital dosimetry (DoseGuard S10; Nuvia Instruments, Dülmen, Germany), as required by German law for radiation protection for technicians and chaperones in the CT room [8]. The minimal radiation detectable by the silicon diode-based dosimeters used was 1 μSv. All dosimeters were set to summation, so they added all radiation dose measurements into a cumulative dose. Dosimeters were placed at 1.5 m height right beside the table at the gantry opening (Dosimeter 1), 1 m away from the gantry opening (Dosimeter 2) and beside the gantry (Dosimeter 3) (Fig. 1).

### Statistical analysis

All measurements were compared using dedicated statistical software (JMP 13 by SAS Institute, Cary, NC; and SPSS Statistics, version 20.0 for Macintosh, by IBM, Armonk, NY). All continuous data were expressed as mean ± standard deviation (SD). The level of significance was set to *P*<0.05. We evaluated the radiation dose for the different locations with a non-parametric one-way repeated-measures analysis (Friedman test). A post hoc Dunn multiple comparison test was performed for group comparison when Friedman test reached statistical significance. All results were Bonferroni-corrected for multiple testing.

## Results

### Phantom scans

First, both protocols were performed using the chest phantom. For Protocol 1 (100-kV chest, non-contrast) no radiation dose was detected on the digital dosimeters after the first scan. Therefore, the scan was repeated. After 10 consecutive scans, using the 100-kV chest, non-contrast protocol, the dosimeter at the gantry opening (Dosimeter 1) showed a total radiation dose of 3.0 μSv and the dosimeter 1 m away from the gantry opening (Dosimeter 2) showed a total radiation dose of 1.0 μSv. Still no radiation was detected on the dosimeter beside the gantry (Dosimeter 3; 0 μSv) (Table 2).

**Table 2:** Cumulative radiation doses (μSv) measured over 10 consecutive scans for every dosimeter^a^ during the scan of the thorax phantom

	Dosimeter 1	Dosimeter 2	Dosimeter 3
Protocol 1^b^	3.0	1.0	0
Protocol 2^b^	2.0	1.0	0

^a^Dosimeter placement is shown in Fig. 1

^b^Scan protocols are specified in Table 1

The 70-kV chest contrast-enhanced protocol (Protocol 2) showed similar results: The dosimeter at the gantry opening (Dosimeter 1) registered 2.0 μSv and the dosimeter 1 m away from the gantry opening (Dosimeter 2) showed 1.0 μSv after 10 consecutive scans. Identical to the 100-kV chest non-contrast protocol, no radiation dose was detected on the dosimeter beside the gantry.

### Patient scans

The radiation dose was measured during 12 pediatric chest CT examinations. Clinical indication for the individual CT scans varied from neuroblastoma staging to unclear pulmonary insufficiency. Mean age of all patients was 1.5±1.4 years, with the youngest child being 2 days old and the oldest 4 years old. The mean age of the 100-kV chest non-contrast protocol cohort was 1.6 years; the mean of the 70-kV chest contrast-enhanced protocol cohort was 1.2 years. Eight children (67%) received 100-kV chest non-contrast protocol and four children (33%) the 70-kV chest contrast-enhanced protocol.

In Table 3, measurements are listed individually for all children. The dosimeter at the gantry opening showed a maximum value of 2.0 μSv in one child during the 100-kV chest non-contrast protocol. In a total of five examinations, for both protocols, 1.0 μSv and 2.0 μSv were registered. In six cases, 1.0 μSv was detected on the dosimeter at the gantry opening. The dosimeter 1 m away from the gantry opening registered radiation only in three examinations (maximum 1 μSv), two times with the 70-kV chest contrast-enhanced protocol. No radiation was detected by the dosimeter beside the gantry in either protocol. As shown in Table 3, overall higher radiation doses with the dosimeter at the gantry opening (Dosimeter 1) were found for the 100-kV chest non-contrast protocol (Protocol 1; 0.75 μSv) vs. the 70-kV contrast-enhanced protocol (Protocol 2; 0.25 μSv). On the contrary, the dosimeter 1 m away from the gantry opening measured radiation only once with the 100-kV chest non-contrast protocol and two times with the 70-kV chest contrast-enhanced protocol.

**Table 3:** Mean values of the two pediatric patient protocols examined

Protocol^a^	Dose^b^ (μSv)			Topogram DLP/CTDI_vol_	Examination DLP/CTDI_vol_	Total DLP
	Dosimeter 1	Dosimeter 2	Dosimeter 3			
Protocol 1	0.75±0.70	0.13±0.40	0	0.49±0.08	3.50±1.10	3.9±1.2
				0.02±0.01	0.17±0.04	
Protocol 2	0.25±0.50	0.50±0.60	0	0.33±0.12	9.40±4.30	9.9±4.1
				0.01±0.01	0.42±0.13	

*CTDI*_*vol*_ volumetric computed tomography dose index, *DLP* dose–length product

^a^Protocol specifications in Table 1

^b^Dosimeter position is shown in Fig. 1

We performed the Friedman test for comparison of the dosimeter positions within the scanner room independent of study protocol. The Friedman test reached statistical significance with *P*=0.034. The post hoc Dunn tests revealed no significant differences among the radiation measuring positions: the dosimeter at the gantry opening vs. the dosimeter 1 m away from the gantry opening, *P*=0.358 (Bonferroni corrected: 1.000); the dosimeter at the gantry opening vs. the dosimeter beside the gantry, *P*=0.066 (Bonferroni corrected: 0.199); or the dosimeter 1 m away from the gantry opening vs. the dosimeter beside the gantry, *P*=0.358 (Bonferroni corrected: 1.000).

The total dose–length product (DLP) of the 70-kV chest contrast-enhanced protocol was more than twice as high as that of the 100-kV chest non-contrast protocol (9.9 mGy‧cm vs. 3.9 mGy‧cm/CTDI_vol_: 0.43 mGy vs. 0.20 mGy).

## Discussion

We were able to show low radiation doses in the phantom scan for all positions around the scanner. After 10 consecutive scans, the highest cumulative radiation dose measured was 3 μSv. For the dosimeter beside the gantry, being nearest to the gantry, even after 10 consecutive scans, no radiation exposure was detected.

These findings from the phantom experiments were confirmed in the clinical setting as extremely low radiation doses (maximum 2.0 μSv), measured during all examinations. Even though these radiation doses were measured after just 1 scan, not 10 in a row, in most cases the legally required dosimeters for chaperones did not register any radiation dose during the CT scan at all — even in close range to the gantry opening. Similar to the phantom scans, at the position next to the gantry no radiation dose was recorded for either protocol, suggesting an overall negligible dose at this spot.

The radiation dose registered right next to the gantry opening and table was higher in the 100-kV chest non-contrast protocol than in the 70-kV chest contrast-enhanced protocol. This might seem counterintuitive because the DLP of Protocol 1 was less than half that of the Protocol 2 DLP (Table 3) and previous studies suggest a significant dose reduction with the use of tin filtering (100-kV chest non-contrast protocol) when compared to conventional low-dose protocols (70-kV chest contrast-enhanced protocol) [4, 9]. The main reason for this discrepancy is most likely scatter radiation. As shown in previous works, scatter radiation depends on tube voltage [10, 11]. The higher the tube voltage, the more scatter radiation is produced. Because the 100-kV chest non-contrast protocol used spectral shaping to filter low-energy photons, the general tube voltage was higher. Thereby, more scatter radiation was produced because of the high-energy photons as the child himself received less radiation by the filtering of low-energy photons. This might be the reason the closest dosimeter (the dosimeter at the gantry opening) registered more radiation in the 100-kV chest non-contrast protocol. What is more, this is most likely the reason for the discrepancy between the phantom scan doses and the actual patient scans. As shown in previous studies, the scatter radiation increases with the scanned volumes’ length [12]. The phantom used for prior testing was relatively short (14 cm) when compared to some of the older children (maximum 18 cm). Thereby, in the patient scans radiation dose was detected in some cases even after only one scan.

The issue of radiation exposure of technicians and chaperones is emphasized by the European Basic Safety Standard Directive “Council Directive 2013/59/Euratom” [13]. The directive as well as the national European legislatures — for example in Germany — reinforce the potential need of dose limit values for chaperones [13, 14]. Our study presents first values needed to establish dose limits for chaperones in modern pediatric CT.

As a result of our findings, we implemented floor marks inside the CT room in our facility for positions of the dosimeter 1 m away from the gantry opening and beside the gantry (Fig. 1) because the lowest radiation dose is expected there. Thereby, and in combination with the use of radiation protection equipment like lead vests and lead glasses, parents and other accompanying adults can safely attend the examination to calm their child during future CT scans. By sharing these promising study results, we hope to see better image quality and less stressed patients in the future.

Our study has several limitations. First, the radiation dose was only measured at one height per position. Measurements might differ when the dosimeter is positioned higher or lower. Nonetheless, in a representative scenario dosimeters would be used in one single height attached to the chaperone’s body, as well.

A second limitation is the measured radiation doses. Because chaperones are always required to wear lead vests during examinations, real-life values would be even lower. But this underlines our theory that chaperones can safely remain in the scanner room during the CT scan without being exposed to relevant radiation doses.

A third limitation is the phantom used for initial testing. The phantom was not previously evaluated as a dedicated pediatric phantom. Nonetheless, the technical specifications are similar to those of evaluated pediatric chest phantoms [15]. Fourth, our findings cannot be extrapolated to other scanner systems or other scanning protocols.

## Conclusion

Overall, we were able to show that taking advantages of a chaperone’s presence to calm a child in the scanner room during pediatric chest CT examinations can be justified without radiation dose objections in modern scanner hardware by choosing the right spot for him or her within the room. According to our results, the position beside the gantry yields the lowest (in our study none) measurable radiation dose.
